# Visualization of microwave near-field distribution in sodium chloride and glucose aqueous solutions by a thermo-elastic optical indicator microscope

**DOI:** 10.1038/s41598-020-80328-8

**Published:** 2021-01-28

**Authors:** Zhirayr Baghdasaryan, Arsen Babajanyan, Levon Odabashyan, Jung-Ha Lee, Barry Friedman, Kiejin Lee

**Affiliations:** 1 Department of Physics, Sogang University, Seoul, 121-742 Korea; 2 Department of Radiophysics, Yerevan State University, 0025 Yerevan, Armenia; 3 Department of Life Science, Sogang University, Seoul, 121-742 Korea; 4 Department of Physics, Sam Houston State University, Huntsville, TX 77341 USA

**Keywords:** Energy science and technology, Materials science, Optics and photonics

## Abstract

In this study, a new optical method is presented to determine the concentrations of NaCl and glucose aqueous solutions by using a thermo-elastic optical indicator microscope. By measuring the microwave near-field distribution intensity, concentration changes of NaCl and glucose aqueous solutions were detected in the 0–100 mg/ml range, when exposed to microwave irradiation at 12 GHz frequency. Microwave near-field distribution intensity decreased as the NaCl or glucose concentration increased due to the changes of the absorption properties of aqueous solution. This method provides a novel approach for monitoring NaCl and glucose in biological liquids by using a CCD sensor capable of visualizing NaCl and glucose concentrations without scanning.

## Introduction

The highly sensitive and stable sensing of electrolytes and biological solutions containing organic molecules has drawn widespread attention in recent years^1–9^. Using microwave measurement techniques, for example, extensive research has been done to develop non-contact and non-invasive monitoring of glucose concentration in blood and biological liquids in medicine^10–15^. In addition, microwave-based similar techniques have been developed and applied to determine electrolyte concentration in aqueous solutions such as NaCl, which play an essential role in living systems^16–18^, as well as chemical^19^, geological^20^, industrial processes^21^, and life science^22,23^. Biofluids such as blood, tears, sweat, urine, and saliva have been primary sources providing disease-specific biomarkers^24,25^. These biofluids are composed of metabolites and minerals (sodium, chloride, potassium, magnesium, zinc, iron, calcium, copper, phosphate). Sensitive single-target sensors with the ability to detect these compounds can reflect health status^25^. Among these compounds, for example, sensing NaCl concentrations in an aqueous solution can provide critical information regarding the water–salt balance in human tissues, hydration levels, and other health diagnoses^1,26,27^. Therefore, the design and fabrication of highly accurate and practice sensors is one of the challenging tasks in modern science. Future healthcare devices should be developed to be comfortable to wear, easy and stable to use, and non-invasive^28,29^.

Many studies have been performed to investigate the effects of the electromagnetic fields on the properties of ionic and organic liquids^30–33^. The main application of the microwave techniques used in the studies is based on the bulk resonators filled with a small volume of liquid for sensing^8,34–37^. Another way to characterize the electromagnetic properties of liquid is a microwave microprobe near-field sensing^38,39^. Electromagnetic coupling between microwave probe and solution strongly depends on the liquid properties and solute concentrations. The reflection/transmission coefficient and/or resonant frequency shift were observed to be correlated with changes in solution concentrations. More recently, computer simulation tools have contributed significantly to the molecular-level understanding of microwave interaction with liquids^40^. Using molecular dynamics (MD) simulation interfaces, researchers investigated the behaviors of polar aqueous solutions upon microwave heating^41,42^, dissociation processes^43^, dynamical couplings between ions and solvent molecules, and the dependence of solvent dynamical properties on ion concentrations^44^.

This study presents a new method for complex aqueous solution characterization by visualization technique. The magnetic microwave near-field (H-MWNF) distribution was visualized around NaCl and glucose aqueous solutions by using a thermo-elastic optical indicator microscope (TEOIM) technique. The experimental results show that the intensities of the H-MWNF inversely correlated with NaCl and glucose concentrations. Easy configuration of the experimental setup and an optical way of visualization of microwave near field without scanning are the advantages of TEOIM method. This technique has some similarity to the colorimetric analysis method^45–47^, where the main difference is that TEOIM provides concentration detection in the microwave range. The colorimetric analysis is a well-known concentration detection method in the optical range where by measuring the light absorption the solute concentration can be measured. The colorimetric analysis method-based measurements are only possible with optically transparent substances, and it is not possible for in-vivo detection of organic solution concentration. The concentration detection in the microwave range provides a possibility to detect the glucose level for in-vivo measurements. Small experimental plastic tubes, the size of which is comparable to the veins, were used as a container for the liquid. One of the real and possible practical ways of glucose level measurement is the measurement of veins, where the main mass of a substance is blood. This study can be a paradigm for future researches, and the TEOIM visualization system can be a practical tool for non-invasive and in-vivo blood analysis. It can also be a practical and useful tool to characterize the dynamic properties of complex liquids, to determine aqueous solution concentration changes and understand the behavior of liquid interaction with the electromagnetic field and microwave heating.

## Experimental setup

**Figure 1:**
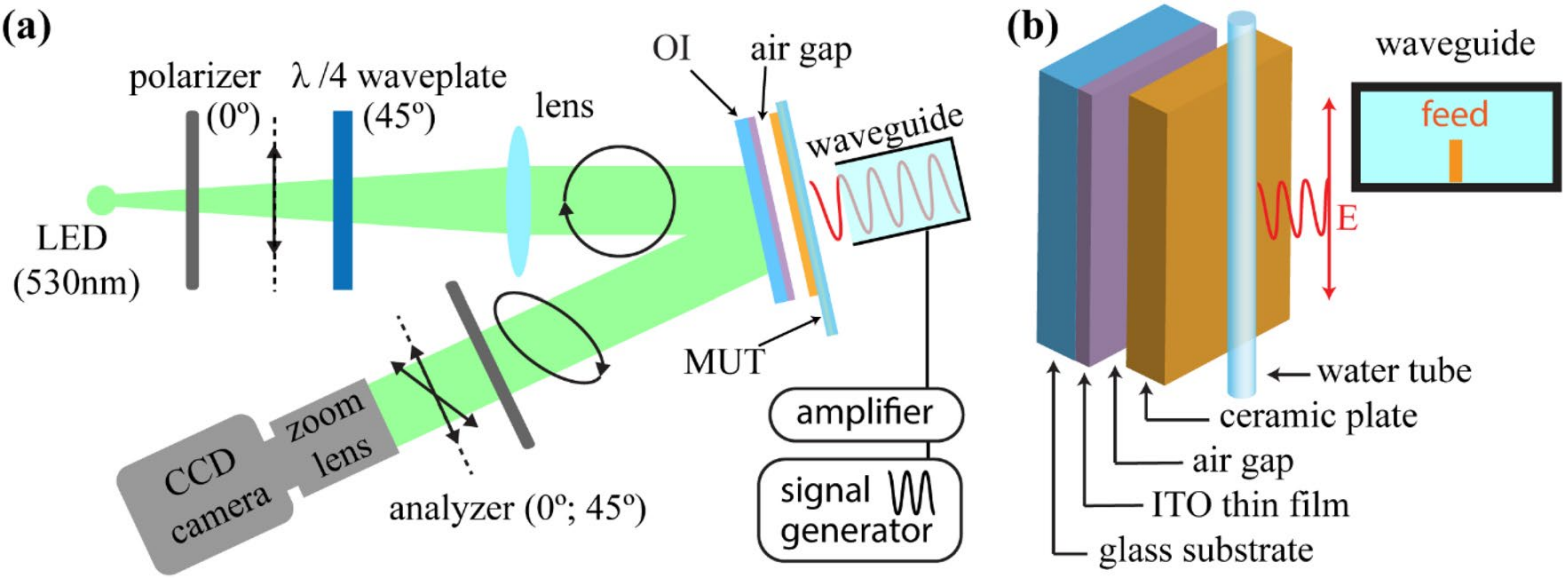
(**a**) Illustration of the visualization system. Probing green light is modulated to be circularly polarized by using a linear polarizer and quarter waveplate. The reflected light passed through the stressed medium changes the polarization to elliptical due to the photo-elastic effect in the glass substrate. Finally, by using the analyzer (linear polarizer sheet) oriented with two different angles, a CCD camera recorded two images of linear birefringence. The generated microwave signal interacts with the MUT which is a plastic tube containing an aqueous solution. The microwave near-field of the aqueous solution excited by the radiated microwave signal interacts with an optical indicator (OI), ITO thin film heats up, and the heat distribution will correspond to the initial microwave near-field distribution around the tube. (**b**) Optical indicator with a water tube, illustration of waveguide and coaxial feed.

Figure 1a shows the experimental setup and sample measurement configuration. The optical indicator (OI) is composed of the glass (Eagle XG glass, 0.7 mm) substrate coated by a 100 nm indium tin oxide (ITO) thin film as a heat absorber (Fig. 1b). For the generation of microwave signals, a synthesized sweeper (HP 83620A) was used. The generated microwave signal at 0 dBm power is amplified up to 35 dBm by using a power amplifier (ZVE-3 W-183 +) and then transmitted by a rectangular waveguide (WR-90, TE mode). The mentioned model of the rectangular waveguide (10.16 mm × 22.86 mm aperture) has recommended frequency range of 8.2–12.4 GHz. Further experiments were performed in the 7–15 GHz range. The radiated electromagnetic wave interacted with the material under test (MUT), changing the shape of field distribution, to be localized around the MUT. The experimental configuration is illustrated in Fig. 1b. The tube was attached to the ceramic plate with 0.38 mm thickness and it was adjusted in the front of the waveguide. Outer and inner diameters of the tube are 1.5 mm and 1 mm, respectively. The distance between the waveguide and material is 10 mm. ITO has high electrical conductivity, and under microwave irradiation a thin layer heats up due to the magnetic field generating surface current in the ITO thin film^48–50^. There was a 1 mm air gap between OI and the ceramic plate to prevent the direct heat transfer from the MUT to the OI. Using the ceramic plate makes it possible to decrease the noise level of the reflected light, to increase the intensity of reflection and to obtain uniform and monochrome view in a visible area of the camera.

The TEOIM technique uses a polarized light microscope system^51–53^ (Fig. 1a). Emitted green light (LED; λ = 530 nm) passes through the linear sheet polarizer (0°) and *λ*/4 waveplate (45°) resulting in circularly polarized light. The incident light is reflected from the OI due to the specular reflection and passes through a linear polarizer (analyzer) (0° and 45°) a second time. Finally, light was recorded by a CCD camera with 1024px × 768px resolution. The setup of the TEOIM visualization system is shown in the supplementary information.

The substrate of the OI is glass which is a thermo-elastic medium, thereby mechanical stress emerges inside the glass during temperature changes. The ITO layer of OI heats up by an applied microwave signal, and the thermal energy from the conductive layer diffuses to the glass. The circularly polarized incident light changes its state to elliptically polarized due to the photo-elastic effect of the glass substrate during the reflection depending on the material characteristics of the medium and orientation of the mechanical stress axis^51^. For image processing two images were measured, both of which were used to calculate the final result by using a custom computer program. We detected the linear birefringent (LB) distribution images with two different analyzer orientation 0° and 45°. These two images, *β*_1_ and *β*_2_ are related to the normal and shear stress distributions of the OI, respectively^51,52^. The initial heat distribution, causing those thermal deformations, was calculated by the following Eq. ^51^:

1 $$q\left( {x,y} \right) = C\left( {\frac{{\partial^{2} \beta_{1} \left( {x,y} \right)}}{{\partial x^{2} }} - \frac{{\partial^{2} \beta_{1} \left( {x,y} \right)}}{{\partial y^{2} }} + 2\frac{{\partial^{2} \beta_{2} \left( {x,y} \right)}}{\partial x\partial y}} \right),$$

where *q* is the density of the heat source, *C* is the constant parameter related to the wavelength of the probing light, and physical properties of the OI. Supplementary information and Ref.^51^ include more detailed information about the TEOIM visualization technique.

Depending on the absorption property of the OI, the visualized field distribution corresponds to the electric or magnetic field. In the case of ITO glass, the film is a uniform conductive layer, and the heat is generated by the alternating magnetic field^48,50^. The heat distribution caused by the generated surface current will be identical to the magnetic field distribution of the incident microwave^51,53^. In the experiment, the H-MWNF distribution was visualized for different solution concentrations in the plastic tube. NaCl and glucose solutions were prepared by weight in concentrations of 0–100 mg/ml.

## Theoretical background

**Figure 2:**
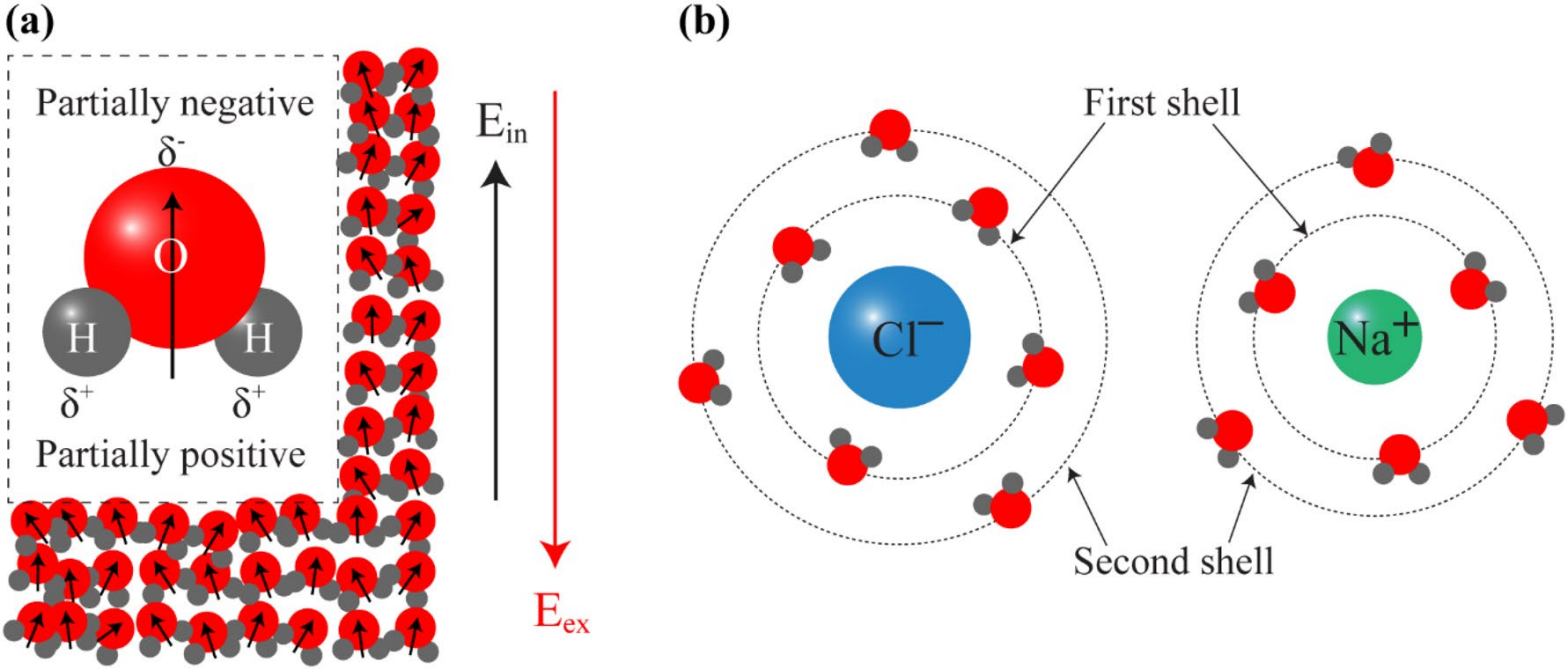
(**a**) Polarization process in the molecular system. Inset shows the water molecule model. (**b**) Bonding structure of NaCl ions with water dipoles. Dashed lines show first and second solvation or hydration shells around ions.

Water is an essential chemical substance in biology and our life. It is made up of one oxygen atom and two hydrogen atoms (H_2_O), and they are bonded with polar covalent bonds (Fig. 2a). The water molecule has a tetrahedral shape, and the side of the oxygen atom is a partially negative charged, due to its high electronegativity. Two hydrogen atoms covalently bond with oxygen because they share the electrons with the oxygen atom. Water molecules have a partially positive charge around the hydrogen atoms^54^. Due to this unique property of water molecule, it is regarded as an ideal solvent for many substances.

The solid NaCl is an ionic compound composed of positively charged sodium (Na^+^) ions and negatively charged chloride (Cl^−^) ions. The attraction between sodium and chloride ions forms an ionic bond. Water as a polar solvent can easily solvate NaCl ions. The ionic bonds between Na^+^ and Cl^−^ ions dissociate due to the interaction of a solute with the solvent. Inside the saline solution, the Na^+^ and Cl^−^ ions start to interact with water dipoles by an ion–dipole interaction (Fig. 2b). Dashed orbits show the first and second solvation shells around ions^55–57^. The bond between two different water dipoles is called a hydrogen bond and this type of interaction is a dipole–dipole interaction. The force of ion–ion interaction is strongest because it involves interactions between formally charged particles, but due to the polarity of water molecules, these bonds are dissociating. The ion–dipole interaction force is less strong because it involves formally and partially charged particles. Finally, dipole–dipole interaction acts between partially charged water dipoles and, thus, it is the weakest^57^. However, water molecules have polarity, and they actively interact with microwaves. Applied external electromagnetic field reorients the dipole moments of molecules. Under the microwave radiation, water molecules tend to align their dipole moments with the alternating external electric field^41^ (Fig. 2a red arrow).

Simultaneously reorientation of dipoles with the same direction is a result of the occurrence of an alternating internal electric field (Fig. 2a black arrow). Figure 2a shows the alignment mechanism of the water dipoles under electromagnetic radiation. The free water dipoles can easily change the orientation of dipole moment under microwave radiation, but dipoles attracted by the sodium and chloride ions have a more stable dipole orientation. Continuous molecular rotation increases the temperature of a liquid and the microwave power density distribution transformed into the microwave heating. All these particular behaviors in the molecular-level are related to the material’s dielectric properties.

Glucose is an excellently soluble compound in the water, but there are no ions, which make glucose aqueous solution a non-electrolyte. Compared with NaCl, glucose (C_6_H_12_O_6_) does not change its chemical chain structure after solvation. This type of solvation is a hydrogen bonding solvation. A glucose molecule has five OH groups (supplementary Fig. S3). In solution, water molecules are hydrogen-bonded to all OH groups. A hydration shell of water surrounds glucose molecules^58^. The supplementary information includes more detailed information about the molecular cluster structure of solved NaCl and glucose.

Electromagnetic plane waves radiated to the dielectric surface penetrate through the material and are partially absorbed and stored inside a material structure depending on the dielectric properties of the material. The volume power density $P_{l}$ in the dielectric medium can be described by the following Eq. ^59^:

2 $$P_{l} = P_{0} e^{{ - \frac{2l}{{D_{p} }}}} ,$$

where $l$ is the distance from the material surface, $P_{0}$ is the power per volume unit at the material surface, $D_{p}$ is the penetration depth. The penetration depth is defined as a distance where the absorbed power density $P_{l}$ decreases *e* times in comparison to the material surface and is expressed by the following Eq. ^31,59^:

3 $$D_{p} = \frac{c}{{\omega \sqrt {2\varepsilon^{\prime } } \sqrt {\sqrt {1 + \left( {\frac{{\varepsilon^{\prime \prime } }}{{\varepsilon^{\prime } }}} \right)^{2} } - 1} }},$$

where $\omega$ is the operating frequency, *c* is the speed of light, $\varepsilon^{\prime }$ and $\varepsilon^{\prime \prime }$ are the real and imaginary parts of the complex dielectric permittivity respectively. The real and imaginary parts of the dielectric permittivity for NaCl and glucose solutions at different concentrations are shown in the supplementary Fig. S4 and S5, respectively. Equations (2) and (3) describe the theoretical model of absorption and power penetration behavior inside the dielectric material. These two equations can clearly describe the microwave heating process.

## Results and discussion

**Figure 3:**
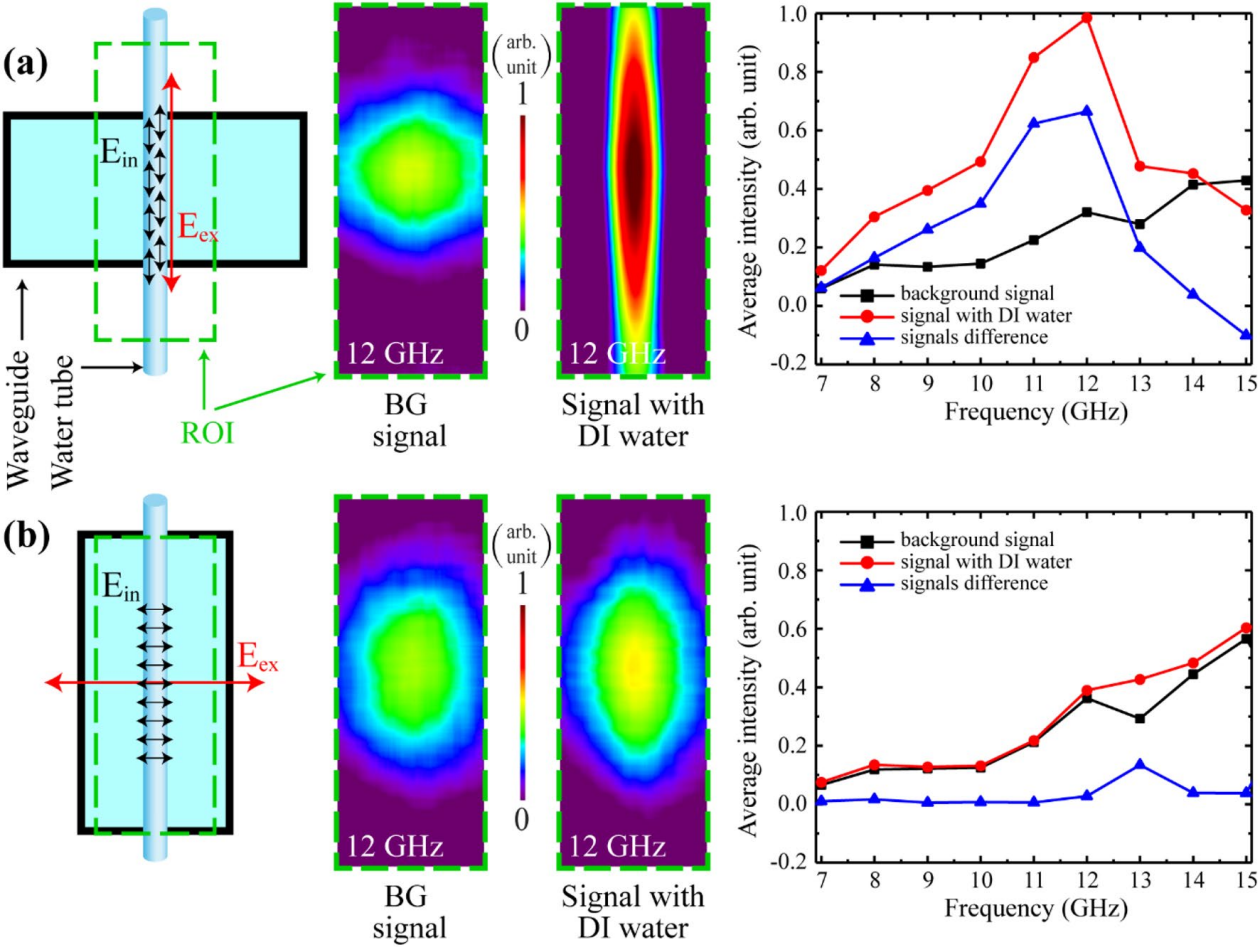
Illustration of the experimental configuration with different waveguide orientations, when (**a**) E-field polarization is parallel to the direction of a tube, and (**b**) E-field polarization is perpendicular to the tube orientation. From left to right shown experimental configuration and green dashed rectangle corresponds to the region of interest (ROI). Next two images show the visualization results of the H-MWNF distributions of the BG signal and for the plastic tube with DI water at the frequency of 12 GHz. The last graph includes information about changes in the average intensity values of the H-MWNF distribution of BG signal (black), the signal with DI water (red) and information about differences of these two signals (blue) in 7–15 GHz frequency range.

In the first step, deionized (DI) water at various frequencies with different experimental configurations was investigated. The field distribution around the liquid tube strongly depends on the polarization of the electromagnetic wave with the orientation of the tube. When the E-field polarization corresponded to the direction of a tube, the electromagnetic field was localized around the tube (Fig. 3a). The shape of the electromagnetic field was changed and aligned with the tube direction. In the other case, when the direction of the tube and E-field polarization were perpendicular to each other, the electromagnetic field distribution was almost unchanged in its shape (Fig. 3b). With this configuration, the liquid did not show a significant effect on a microwave radiation pattern. Essentially, the effect is negligible. In the entire liquid system, the water dipoles tend to align with an electric field direction. Rapid field direction reversals of the electric field produced internal alternating electric field due to dipoles in a similar motion when the liquid was exposed to electromagnetic radiation. Significant impact on the external electromagnetic waves can emerge only when the internal alternating electric field is stronger. In the case of Fig. 3a configuration, the internal electric field corresponding to the tube direction would be stronger, because a sufficient number of dipoles might oscillate along a tube direction. In Fig. 3b configuration; however, internal electric field and tube direction are perpendicular to each other. Dipole rotation direction is oriented to the cross-sectional direction of the tube.

The intensity of the internal electric field distributed along a tube and did not correspond with the tube orientation. That might be the potential reason for detection of the negligible effect on a background (BG) signal. In Fig. 3a, signal intensity with DI water is significantly higher compared with BG signal, because the localization effect of the radiated microwave signal distributed around the plastic tube. Moreover, the alternating internal electric field can generate secondary electromagnetic radiation from the liquid due to the elongated distribution of the liquid. In a perfect conductor, there are free electrons, and their alternating flow can generate electromagnetic radiation. In this system, DI water also contained free oriented dipoles. Alternating external electric field generates alternating internal electric field inside the DI water. This internal electric field surrounded by the alternating magnetic field, results in the visualized images which show the microwave magnetic field distribution in the near zone of the tube. To reiterate, water excited in the container due to the external microwave radiation is surrounded by an intense magnetic field because of the dipolar electric field inside the water. The H-MWNF distribution was measured using the TEOIM visualization system. The graphical behavior for each configuration shows that the large difference of averaged intensities between the visualization results of DI water and the results of microwave BG signal emerges at 12 GHz operating frequency (Fig. 3a, blue line). The reason behind a visualized high intensity at 12 GHz excitation is an experimental configuration limited by the optimal operating frequency range of the waveguide (8.2–12.4 GHz), OI, and peculiarity of the coupling between liquid sample and microwave irradiation. An additional experiment was performed to substantiate this approach. In this experiment tubes with three different inner diameters (0.5 mm, 1 mm, and 2 mm) were used. All results of the additional experiments are presented in the supplementary information. Therefore, for further measurements, 12 GHz was chosen as an optimal frequency for the investigations of aqueous solutions, and E-field polarization aligned corresponding with tube orientation (Fig. 3a). In further experiments, the inner diameter of the tube container was 1 mm.

**Figure 4:**
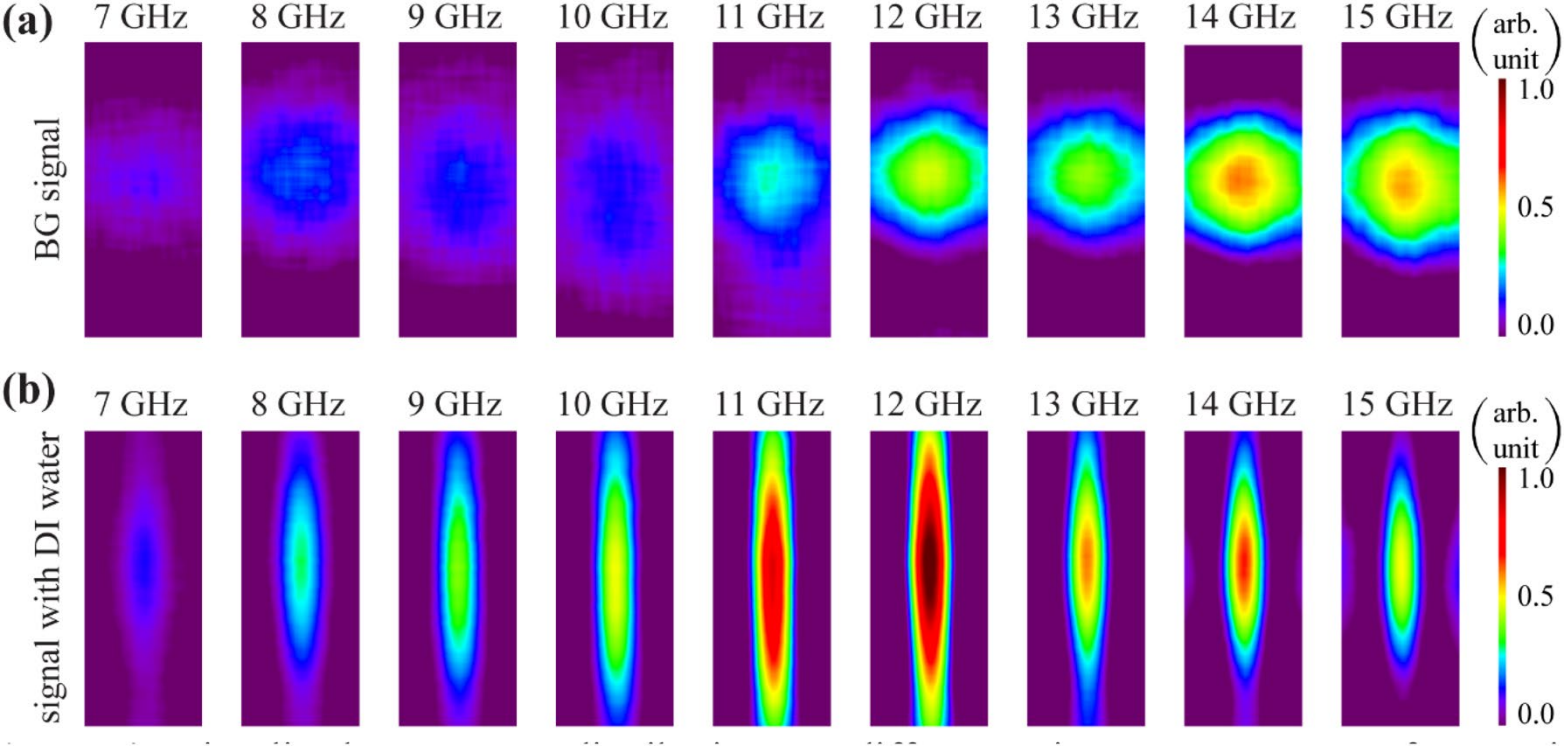
Visualized H-MWNF distributions at different microwave exposure frequencies when the plastic tube was empty (**a**) and filled with DI water (**b**). Size of each image is 20 mm × 8 mm.

Figure 4 shows representative images measured at various frequencies. Figure 4a corresponds to the H-MWNF distribution of a BG signal when the plastic tube was empty. The results show that the H-MWNF pattern without DI water has a circular shape, which refers to the fundamental mode of the rectangular waveguide used as a source. The series of images in the Fig. 4b shows the H-MWNF distribution for a plastic tube filled with DI water. In this case, DI water strongly interacts with microwave radiation. Due to the interaction, microwave radiation is localized around the tube and changes its shape, aligning itself along the tube axis. The subsequent simulation was performed to show the localized field around the plastic tube filled with DI water. From these images the average characteristic values were calculated for various frequencies. The average intensity data in Fig. 3a were estimated based on the visualized images (2D matrix) shown in Fig. 4a,b.

**Figure 5:**
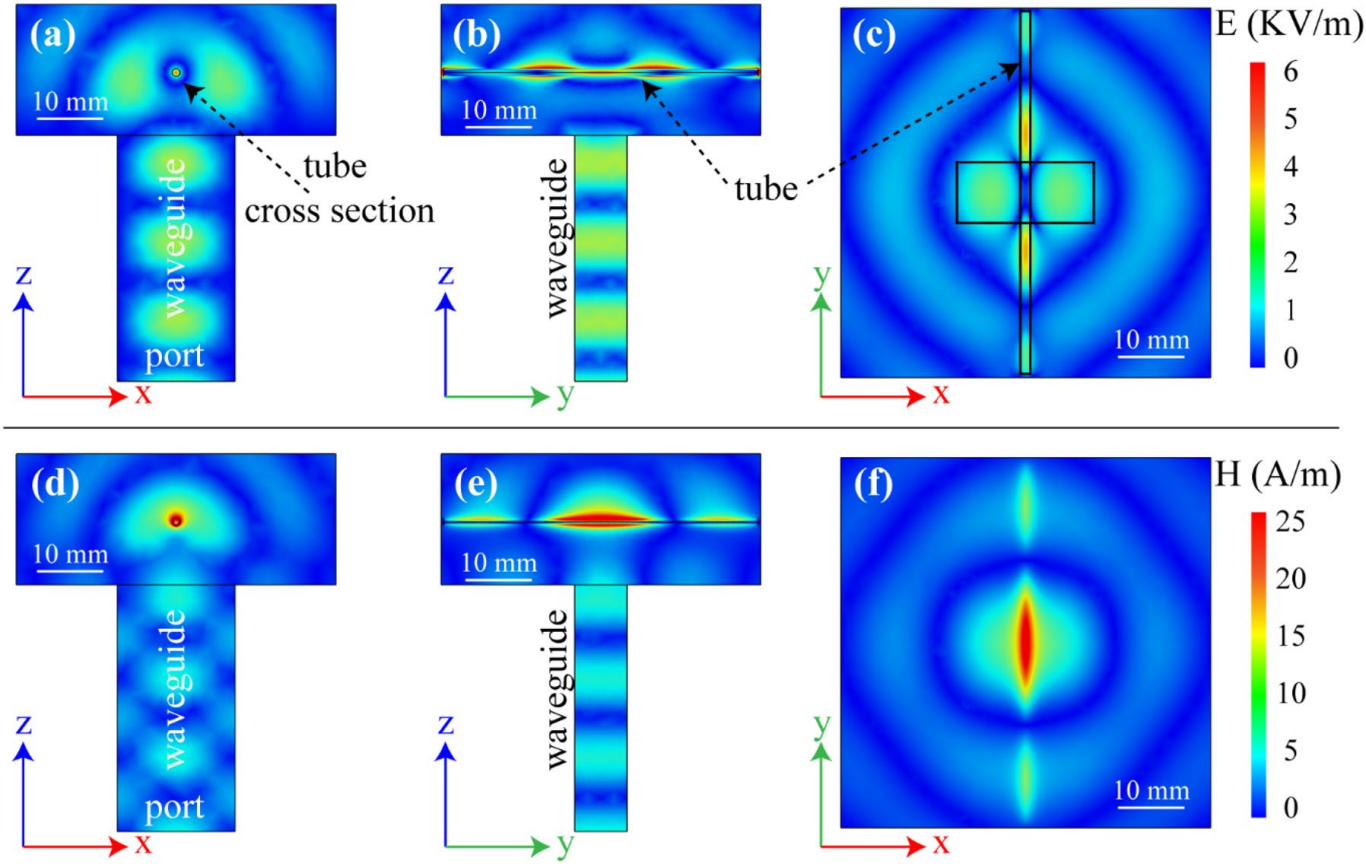
Simulation results for (**a**–**c**) electric and (**d**–**f**) magnetic field distribution with three different projections at 12 GHz.

Electric and magnetic field distributions around the tube were simulated through a 3D full-wave numerical analysis using the COMSOL Multiphysics software based on the finite element method. The input power of the rectangular waveguide was set 3.1 W (~ 35 dBm). The distance between the tube and the waveguide is 1 cm which is identical to the experiment. The system is enclosed by a box with the scattering boundary conditions applied to its walls. These boundary conditions with large enough box sizes (triple of operating wavelength) prevent the influence of the back-scattered waves from the boundaries and imitate free space. In the system, mutual coupling occurs between open-ended waveguide and the plastic tube filled with DI water. In Fig. 5a–f, the simulation results for E-field and H-field distributions are shown with three different projections, at 12 GHz. Figure 5f shows a pattern of H-field distribution around the tube which is perfectly matched with the experimentally visualized field distribution (Fig. 4b). Again, H-MWNF field was localized around the MUT (Fig. 5d,e).

**Figure 6:**
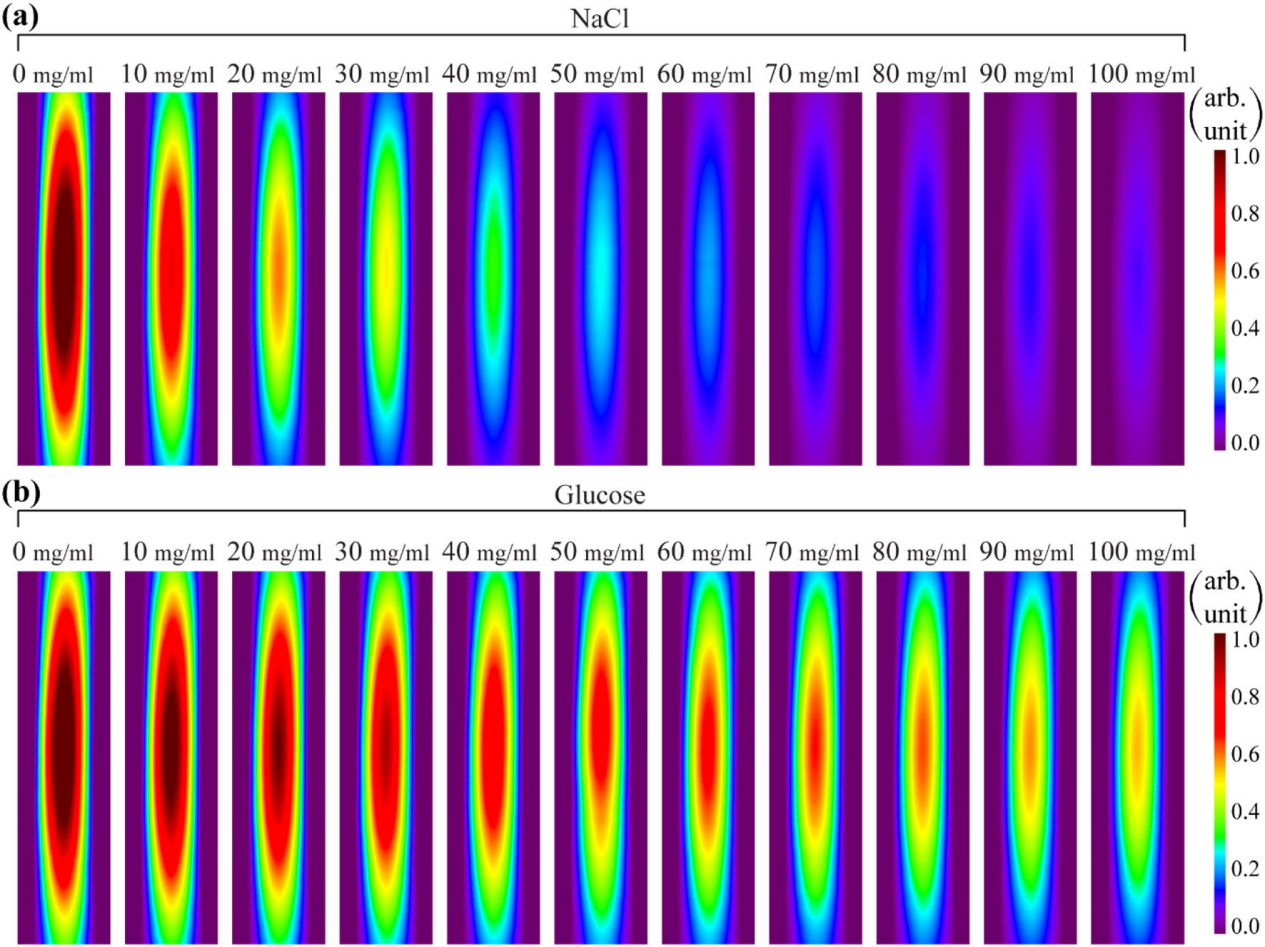
H-MWNF distribution images for (**a**) NaCl and (**b**) glucose aqueous solutions with 0–100 mg/ml concentration at 12 GHz visualized by TEOIM. Size of each image is 20 mm × 5 mm.

Experimentally visualized images for NaCl and glucose aqueous solutions in the concentration range of 0–100 mg/ml are shown in Fig. 6. These results carry the visual information about the distribution of the electromagnetic field around the plastic tube containing NaCl and glucose aqueous solutions with different concentrations. As solution concentrations increased, the intensity of the H-MWNF in the solution decreased in both cases.

**Figure 7:**
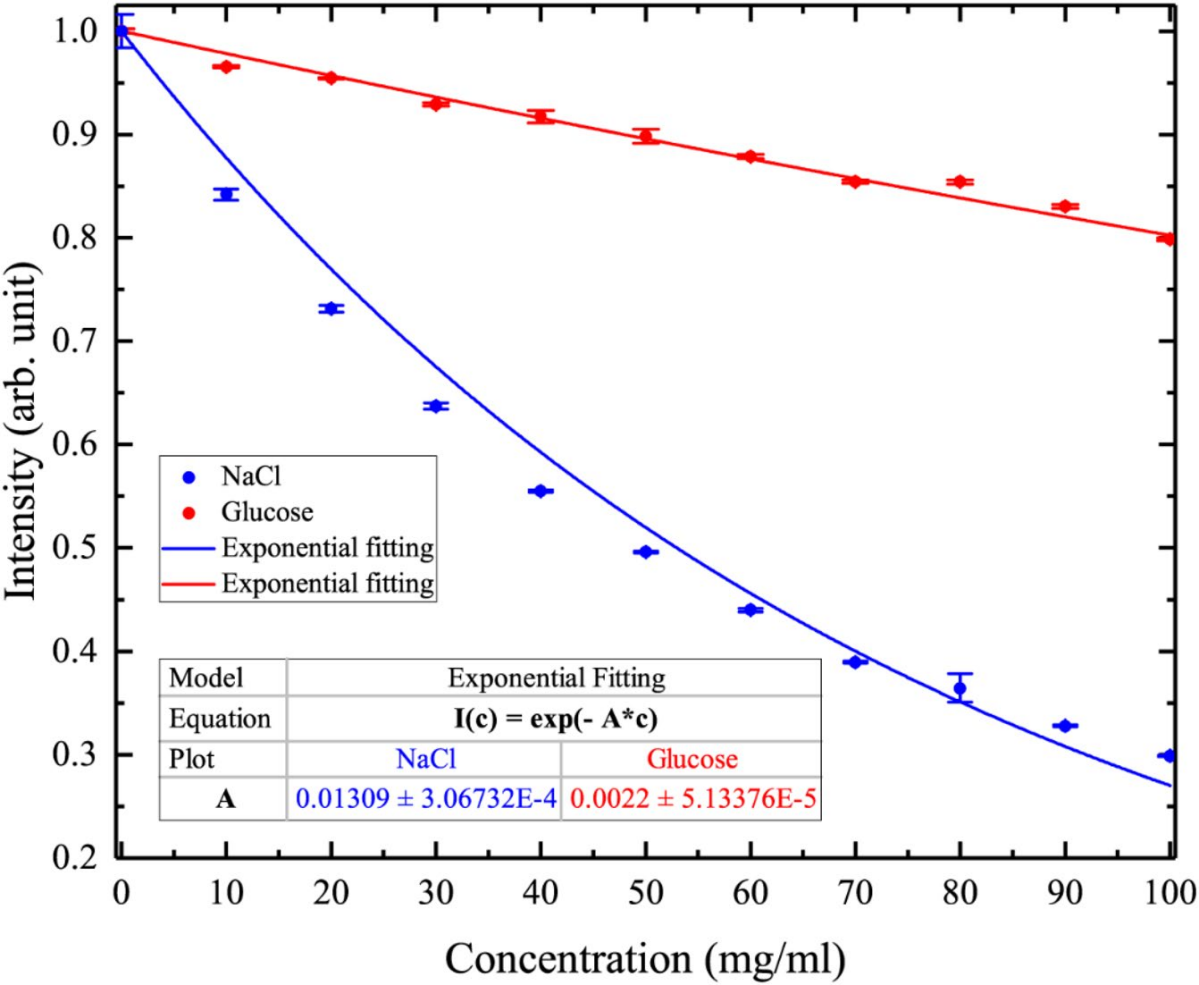
Graphical behavior of averaged intensity for H-MWNF distribution depends on the NaCl and glucose concentrations in aqueous solutions ranging from 0 to 100 mg/ml at 12 GHz. Error bars represent mean ± standard deviation of five independent experiments for each concentration. Solid line shows the exponential fitting of the experimental data.

**Figure 8:**
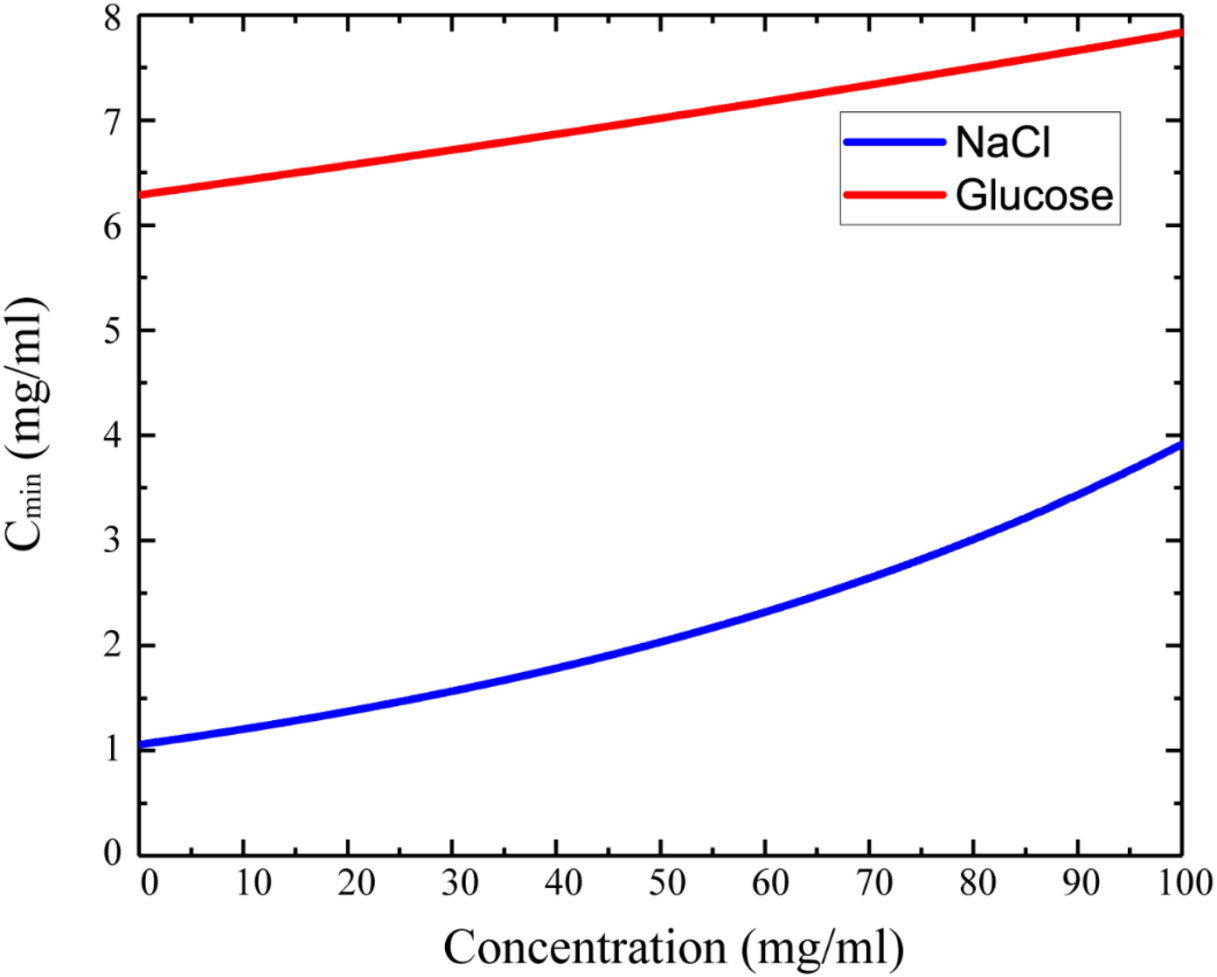
The calculated minimum detectable concentration as a function of NaCl (blue line) and glucose (red line) concentrations in aqueous solutions.

Figure 7 shows the intensity changes of the H-MWNF distribution images for 0–100 mg/ml concentration range with serial step increases of 10 mg/ml at 12 GHz. The experiment was repeated five times for each NaCl and glucose concentration. Each final image is the average of 3000 images taken by the CCD camera. Red points show the arithmetic mean of five-time measurements, and error bars correspond to the mean ± standard deviation of independent experiments. Based on this experimental data $C_{min}$ (minimum detectable concentration) was calculated for NaCl solutions by the following equation:

4 $$C_{min} = \left| {\Delta E_{max} \left( {\frac{dI\left( c \right)}{{dc}}} \right)^{ - 1} } \right|,$$

where $\Delta E_{max}$ has chosen as a maximum fluctuation from all concentration measurement corresponding to the margin of error. The $I\left( c \right)$ is the intensity function of the image of the solution depending on the $c$ concentration. Figure 8 shows the minimum detectable concentration ($C_{min}$) change as a function of solute (NaCl and glucose) concentration in solution. The $C_{min}$ increased as shown in the graph and the sensitivity of the system decreased^38^. The normal blood glucose level is 0.72–1.44 mg/ml, whereas the pathophysiological range is 0.36–5.4 mg/ml^60^. The normal level of NaCl in the blood is 9 mg/ml (0.9%)^61^. As follows from Fig. 8, the TEOIM technique does not have enough sensitivity to detect blood level glucose concentration, but this is not the limit of sensitivity. The system can reach higher sensitive detection and faster characterization of the material.

One of the ways to get more accurate data and to decrease error level is an advanced indicator such as metamaterial-based indicators. In addition, some microwave structures integrated with the tube could be also considered as a second way to improve the accuracy of the sensing technique. However, in the case of NaCl, the sensitivity of the system is higher and is able to distinguish NaCl or another ionic compound in biofluids with a detection resolution of about 1 mg/ml. In particular cases, this novel technique can be a useful tool in a research environment.

Experimentally visualized H-MWNF distribution shows the highest intensity in the case of DI water where the quantity of free dipoles is a maximum (Figs. 6 and 7). As of sodium chloride ions (Na^+^ and Cl^−^) or glucose concentrations in the solution increase, the number of free water dipoles decrease, resulting in the H-MWNF distribution with lower intensity. The relative permittivity changes depend on the ionic concentration influence on the absorption property of NaCl solutions. Absorption behavior theoretically can be described by the Eqs. (2) and (3). In this model, the liquid absorption changes exponentially, being saturated for a high concentrated solution. Visualized H-MWNF intensity distribution would relate to the absorption property of the solution. The relationship of the intensity versus NaCl concentration is not linear, and with the higher solution concentration the curve becomes saturated (Fig. 7).

Alternatively, a possible microscopic reason for the intensity decreasing behavior is that the second solvation shell of ions has higher stability at low concentrations^40^. By increasing the solution concentration in the second shell probability of ion–dipole attraction decreases and the process is saturated. From Fig. 7, it turns out that in the case of NaCl, the intensity decrease is bigger. The reason for the phenomenon is that NaCl and glucose have different solvation mechanisms. In NaCl solution the ion–dipole interaction is stronger and more stably bonded, whereas in glucose solution the hydrogen bonds between glucose and water molecules are relatively weak^40,62,63^. Upon microwave excitation, in the saline water, the mobility of the free water molecules is higher. Additionally, in this system, there are ionic current flows, but ion dipoles pairs are better bonded, especially in the first solvation shell. In comparison to Na^+^ and Cl^−^ ions, the glucose molecule is bigger and has a complex chain structure. This solution is a dielectric where glucose molecules would immobile. However, the hydrogen bonds change more dynamically because of a weak attraction.

The microwave effect on the solution in all cases has the highest intensity when the liquid sample is DI water without mixing of any substances. In this case, all water dipoles are free, and they respond to microwave radiation intensively. This study also presents the quantitative measurement of H-MWNF distribution of NaCl and glucose concentration in an aqueous solution separately. However, the present sensing technique is limited to distinguish certain substances in mixture solutions specifically. Mixing several substances will decrease the field intensity, but in this case, it's hard to distinguish the effect of the specific substance separately. Despite the limitation, this technique would be applicable to measure changes in the blood glucose concentrations of diabetes patients whose other blood solutes are relatively stable. As reported in other researches^39,64,65^, the response of the mixture has an additive effect and is the sum of the influences of both substances (the impact of NaCl was much higher).

## Conclusions

The effects of microwave radiation on NaCl and glucose solutions with different concentrations were investigated and visualized by using TEOIM technique. Microwave interaction depended on the electromagnetic field polarization and liquid orientation. With a fixed orientation of the liquid tube and waveguide, the H-MWNF distribution intensity dependence on the NaCl and glucose concentration variation was investigated at 12 GHz. The experimentally visualized H-MWNF showed a quite similar distribution with the simulated result. As the NaCl or glucose concentration increased from 0 to 100 mg/ml, the intensity of the H-MWNF distribution decreased, and then saturated for high concentration solutions. This new optical method can be applicable to advanced non-contact and non-destructive testing approaches for the electromagnetic property of aqueous solutions, and to determine solute concentration changes in aqueous solutions for ionic and non-ionic complex substances.

## Supplementary Information

Supplementary Information 1.

Supplementary Information 2.

Supplementary Information 3.
