# Supplementary material for: Visualization of microwave near-field distribution in sodium chloride and glucose aqueous solutions by a thermo-elastic optical indicator microscope
Source: Sci Rep. 2021 Jan 28;11:2589. doi: 10.1038/s41598-020-80328-8 (PMC7843988; doi:10.1038/s41598-020-80328-8)
Supplement: Supplementary file 1 — Supplementary Information 1. [file 41598_2020_80328_MOESM1_ESM.pdf]

Supplementary information

Visualization of microwave near-field distribution in sodium chloride and glucose aqueous solutions by a thermo-elastic optical indicator microscope

Zhirayr Baghdasaryan, Arsen Babajanyan, Levon Odabashyan,
Jung-Ha Lee, Barry Friedman, Kiejin Lee

Supplementary Information 1

Figure S1 shows the configuration of the TEOIM visualization system. All measurements were implemented in the dark environment to prevent alternative light noises.

(a)

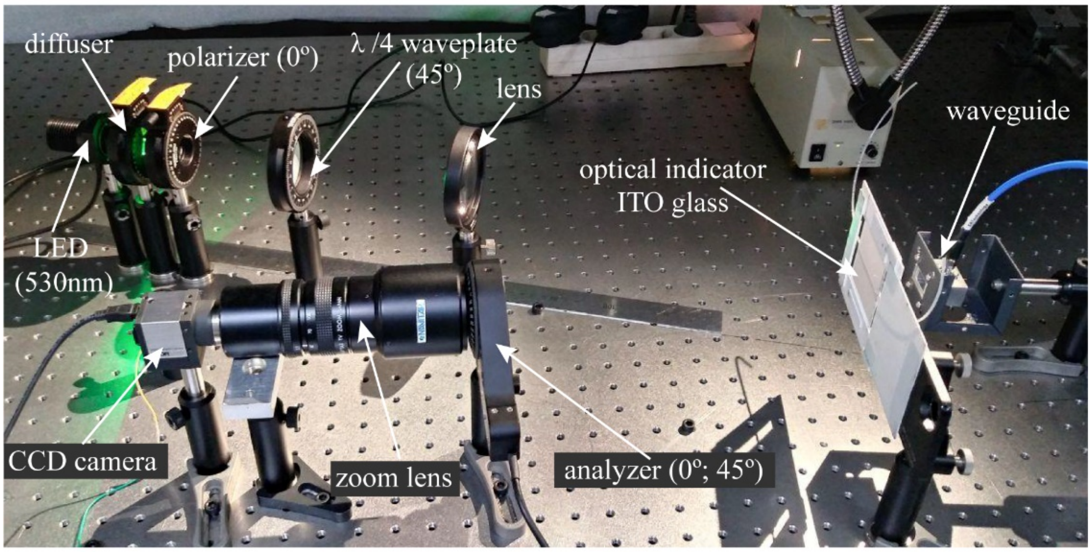

(b)

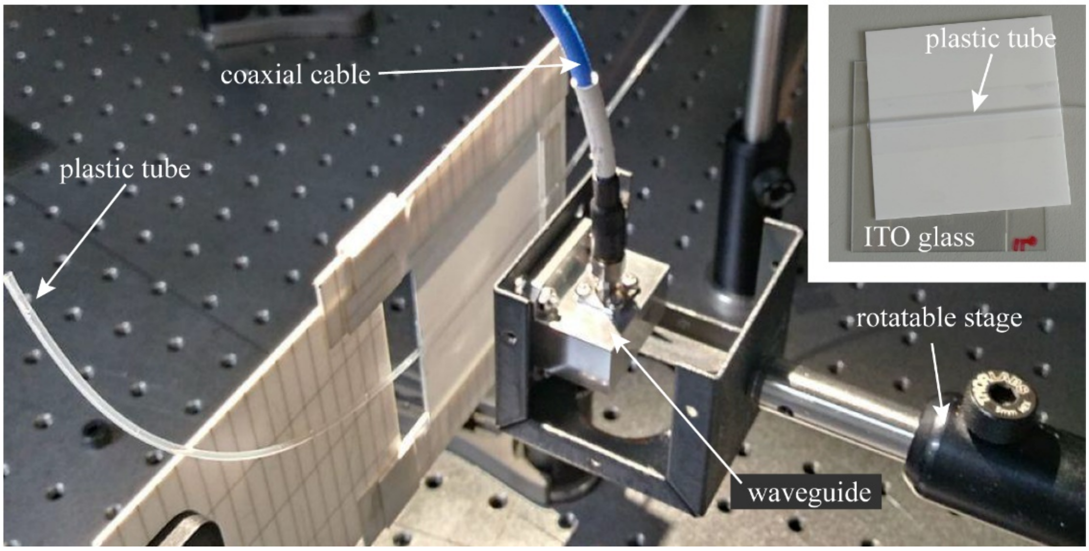

Supplementary figure S1. (a) The Photograph of the TEOIM visualization system and (b) experimental adjustment for sample holder.

Figure S2 (a) shows the sequence of visualization steps. The dielectric property of the liquid also has temperature dependence. For that reason, after turning on the microwave signal, we waited about 4 seconds for temperature saturation. This visualization is a stationary state measurement method.

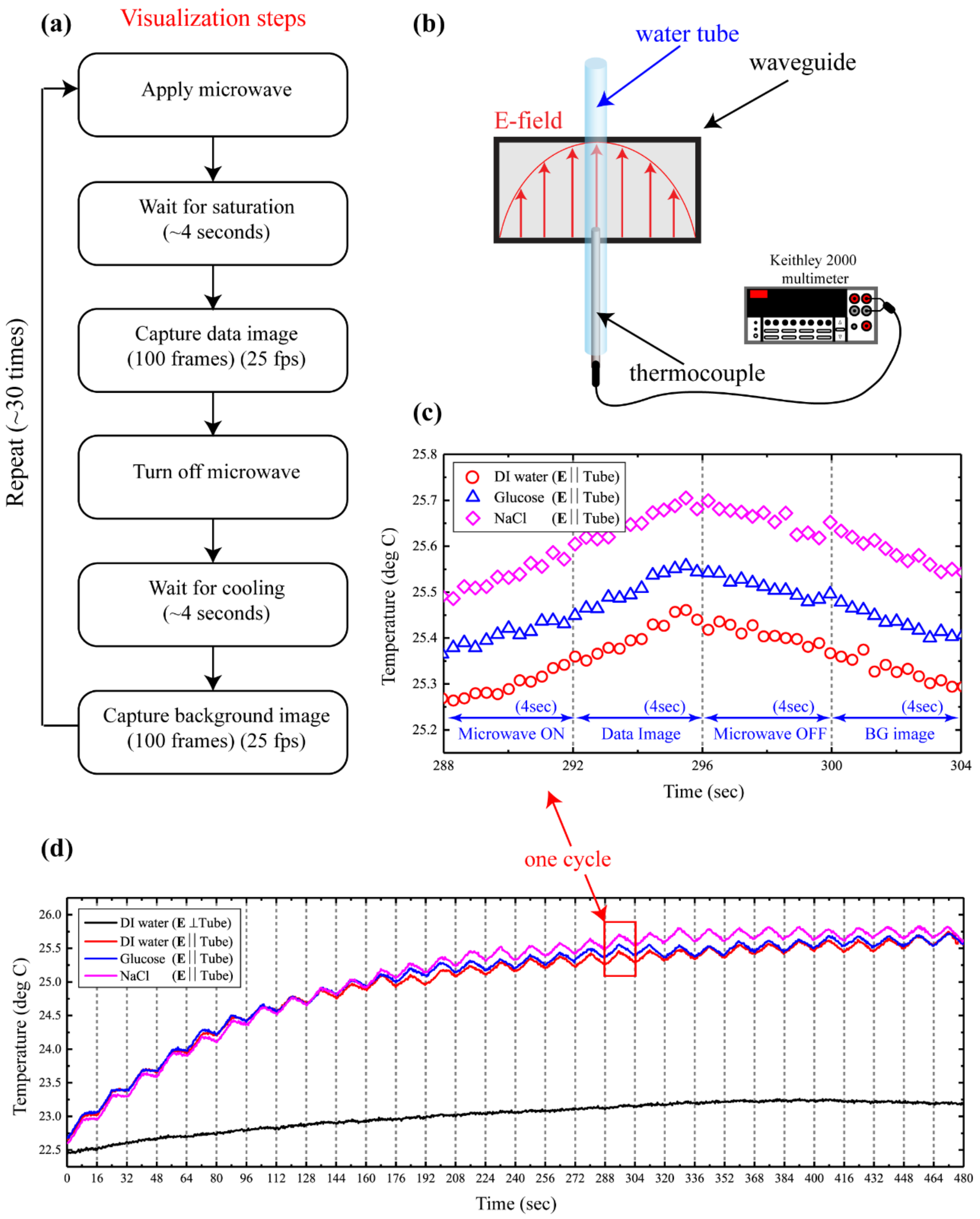

Supplementary figure S2. (a) The schematic of the visualization process. (b) Illustration of an experimental configuration for measuring the temperature of DI water during microwave excitation. (c) Temperature variation of DI water during one cycle of the experimental loop. (d) Temperature variation of aqueous solutions during all the experimental process. The black line is a particular case where the E-field was perpendicular to the tube orientation. The red, blue, and pink lines show the temperature variation in solutions of DI water, glucose (50 mg/ml),

and NaCl (50 mg/ml), respectively, when the polarization of the E-field was parallel to the tube.

Figure S2 (b) illustrates a liquid temperature measurement configuration setup, where the variation in water temperature during microwave excitation was investigated. The experimental conditions were identical to those in the visualization process. The temperature was measured directly inside the liquid by using a thermocouple. Real-time data were registered using a Keithley 2000 multimeter under microwave excitation. Figure S2 (c) shows the temperature dependence on time of one experimental cycle, which takes 16 seconds. By following the Fig. S2 (a) sequence, there is a 4 second camera waiting period for microwave thermal equilibrium before taking the data images. During that stage, the water temperature was slightly increased, and in the next 4 seconds, the camera took 100 data images (25fps). After this process, the microwave signal was turned off, allowing the water to cool down. Again for 4 seconds, the camera took the background images for final subtraction. These cycles were repeated approximately 30 times to obtain clear images. Figure 2 (d) shows the water temperature dependence on the time during the whole measurement processes, which takes 8 minutes. The number of cycles could be reduced by increasing the microwave power and replacing the indicator with a more sensitive one. By increasing the microwave power, the fewer number of cycles (~10 times) would show the same behavior, image resolution, and quality. In this case, the time of the experiment can be reduced drastically. Figure S2 (d) black line shows that the water inside the tube did not heat appreciable. In this particular case, the waveguide was rotated 90 degrees, the direction of the E-field was perpendicular to the orientation of the tube, and the microwave effect was negligible. Otherwise, the temperature of aqueous solutions was increased about 3℃ when the polarization of the E-field was parallel to the tube. The red, blue, and pink lines show the temperature variation in solutions of DI water, glucose (50 mg/ml), and NaCl (50 mg/ml), respectively. Experiments showed that DI water, glucose, and NaCl, solutions produced very similar heating behaviors when they was excited by microwave radiation. The thermal influences on the visualized results were ignored during the imaging, because the temperature variations during the imaging were less than 3℃. Similarly the influence of the latter on the dielectric properties of aqueous solutions can be neglected[1–4]. As mentioned in the manuscript, there was a 1 mm air gap between the OI and the ceramic plate to prevent the direct heat transfer from the MUT to the OI. Finally, it was considered that the OI heats up only due to the magnetic near-field. The thermal fluctuation of the MUT does not have any effect on the visualized magnetic field distribution.

Supplementary Information 2

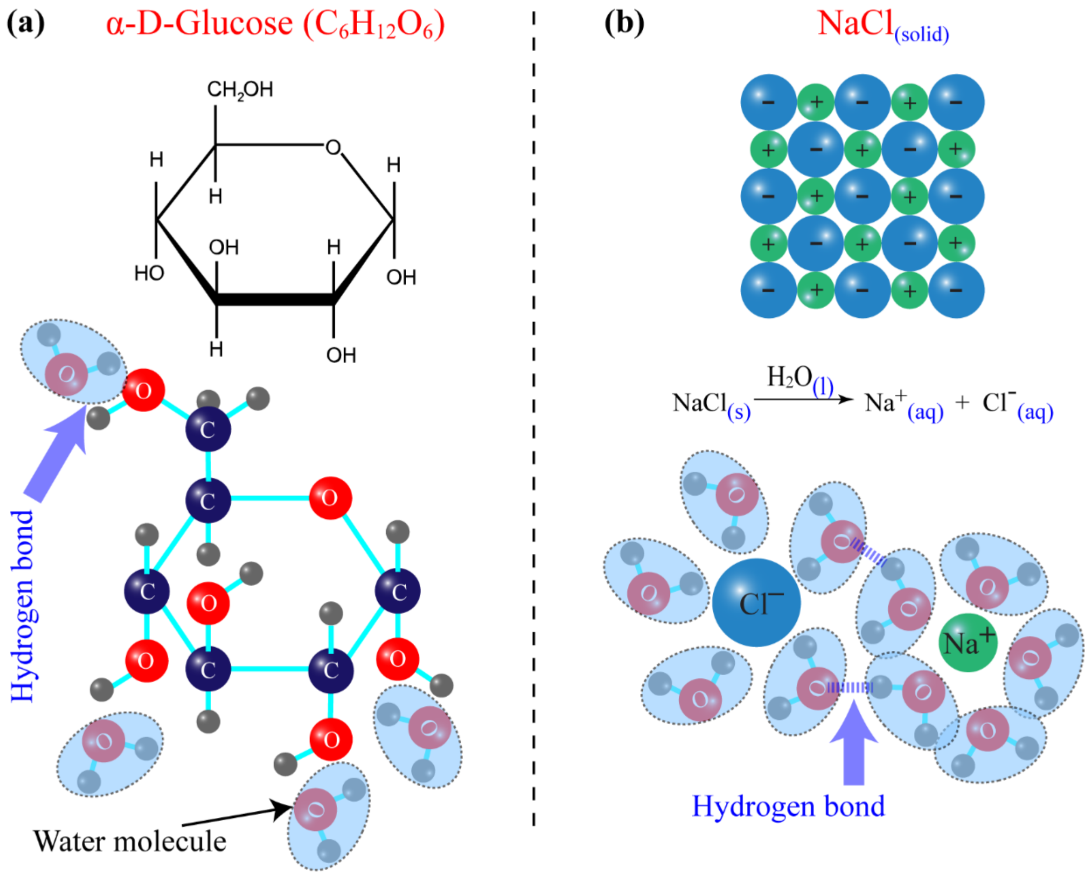

Supplementary figure S3. (a) Cyclic forms of D-glucose molecule with hydration mechanism. (b) The crystal system of a NaCl and illustration of the saline water bonding structure in the first solvation shell.

The main reason of solvation is an interaction between solvent and solute molecules. The forces in solvation are ion-dipole and hydrogen bonding attractions. In the organic solution like a glucose water solution, the interaction emerges between OH groups of the glucose molecules and water polar molecules (Fig. S3 (a)). In the case of NaCl water solution, there are strong ion-dipole attractions between the ions and the water molecules (Fig. S3 (b)). The Na^+ ions attract the negative oxygen atoms of water, and Cl^- ions attract positive hydrogen atoms of water. The ions become surrounded by a solvation shell of water molecules. In the first solvation shell, opposite charges get closer to each other and the system is more stable. This solvation stabilizes the ions.

Supplementary Information 3

The dielectric properties of NaCl and glucose aqueous solutions describe the interaction principles between liquid and microwave radiation[5]. The complex relative permittivity is defined by the following equation:

$$\varepsilon = \varepsilon' - j\varepsilon'', \tag{1}$$

where ε' is the real part of complex electrical permittivity and is related to the ability of the material to store electromagnetic energy in the material structure. The imaginary part of complex permittivity ε'' caused by the conductivity characterizes the energy loss when a microwave passes through the liquid[5,6]. Dielectric constant and loss tangent are a function of microwave frequency and solute concentration, and these dependencies are shown in Fig. S4 and S5 for NaCl and glucose solution, respectively. Those were calculated by using the Debye relaxation model

$$\varepsilon' = \varepsilon_\infty + \frac{\varepsilon_S - \varepsilon_\infty}{1+\omega^2\tau^2}; \tag{2}$$

$$\varepsilon'' = \frac{(\varepsilon_S - \varepsilon_\infty)\omega\tau}{1+\omega^2\tau^2},$$

where $\varepsilon_\infty = 8.722$ is the permittivity in the high frequency limit, $\varepsilon_s = 81.015$ is the static, low frequency permittivity, and $\tau = 8.776ps$ is the relaxation time[6,7]. Loss tangent ($\tan\delta$) is another electromagnetic parameter of a material and is defined as

$$\tan\delta = \frac{\varepsilon''}{\varepsilon'} \tag{3}$$

and demonstrates how well a material convert stored energy into heat. Fig. S4 (b) and Fig. S5 (b) shows loss tangent dependence on the NaCl and glucose solutions concentrations in 7-16 GHz frequency range, respectively.

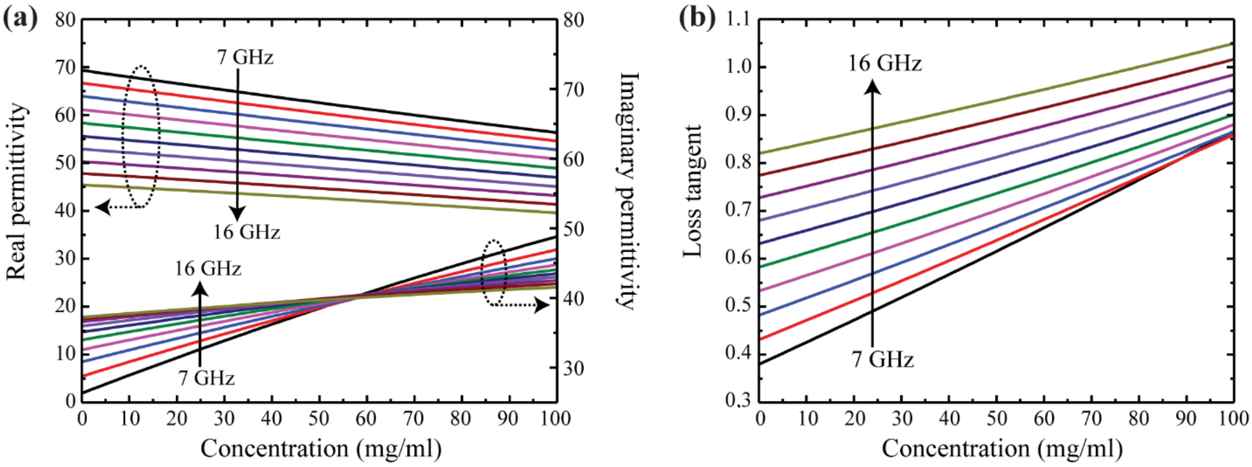

Supplementary figure S4. (a) Calculated real and imaginary parts of the complex relative dielectric permittivity of NaCl aqueous solution as a function of NaCl concentration in 7-16

GHz frequency range. (b) The dependence of the electric loss tangent on NaCl concentration (0-100 mg/ml) in the 7-16 GHz frequency range.

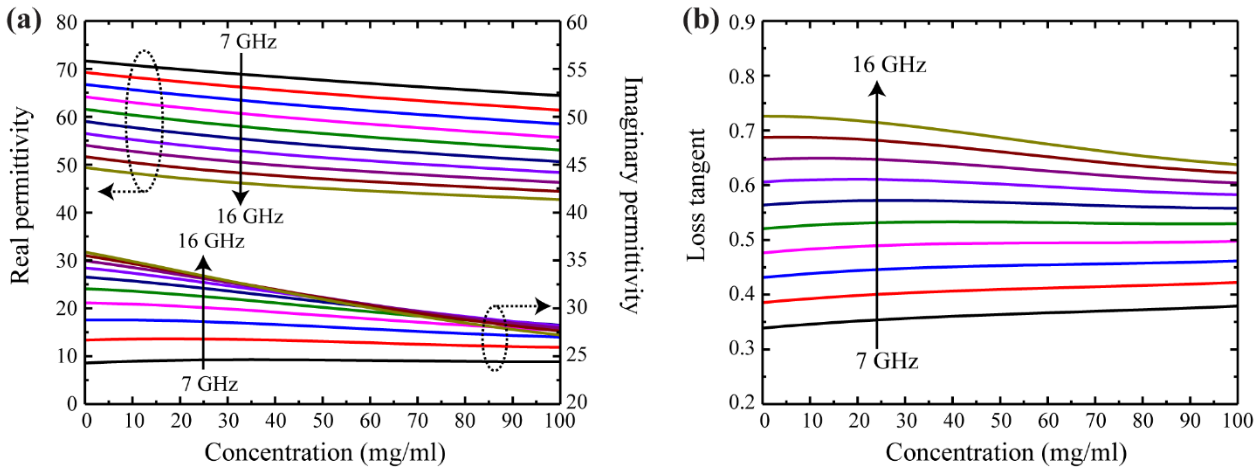

Supplementary figure S5. (a) Calculated real and imaginary parts of the complex relative dielectric permittivity of glucose aqueous solution as a function of glucose concentration in 7-16 GHz frequency range. (b) The dependence of the electric loss tangent on glucose concentration (0-100 mg/ml) in the 7-16 GHz frequency range.

Supplementary Information 4

An additional experiment was done to understand the coupling behavior of the water tube container with microwave radiation. The WR-90 (TE_{10} mode) rectangular waveguide was used as a microwave transmitter, which is a metallic cavity with dimensions of the aperture of 10.16 mm × 22.86 mm. This waveguide is more optimal to use in the frequency range from 8.2 GHz to 12.4 GHz. There is no strong limitation of frequency, but when the generated microwaves cross that range the symmetric pattern of the microwave radiation is destroyed for higher frequencies. To reiterate, when exceeding this frequency range, the wavelength of the electromagnetic wave isn't reciprocal to the linear dimensions of the cavity, and the symmetric pattern of the microwave field distribution is disrupted. The images at 14 GHz and 15 GHz (Fig. S6 (column (a)) show that the microwave pattern is closer to an ellipse unlike the circle at lower frequencies. By increasing the microwave frequency up to 15 GHz, the intensity area separating two parts and secondary wave modes start appearing in the cavity, which means that the mode is TE_{20} henceforth. The experiments therefore indicate using a 7-15 GHz range with this configuration.

The results show that the coupling between liquid and microwaves is different for the different tube diameters. Fig. S6 shows the visualized H-MWNF distribution images using tubes with 0.5 mm (column (b)), 1 mm (column (c)), and 2 mm (column (d)) diameters. The response of the small tube is higher when the water is exposed to a higher microwave frequency (Fig. S6 column (b) 13 GHz, 14 GHz, 15 GHz and Fig. S7 red line), and for the large tube diameter the response is higher at low frequency (Fig. S6 column (d) 7 GHz, 8 GHz, 9 GHz and Fig. S7 pink line). The most effective response is shown for the tube with a 1 mm diameter, which shows a noticeable difference compared with the 0.5 mm and 2 mm tubes. For the main experiments this inner diameter of tube was used (Fig. S6 column (c) 11 GHz, 12 GHz, 13 GHz and Fig. S7 blue line). Note that even in this simple configuration, there are many factors that change the microwave coupling and heating efficiency of the aqueous solution. Experimentally it was clarified that the liquid contained in the small diameter tube shows a more effective microwave response when the wavelength of the microwave radiation is shorter (frequency is higher). When the tube diameter is larger, the microwave response is stronger at longer wavelengths (lower frequency).

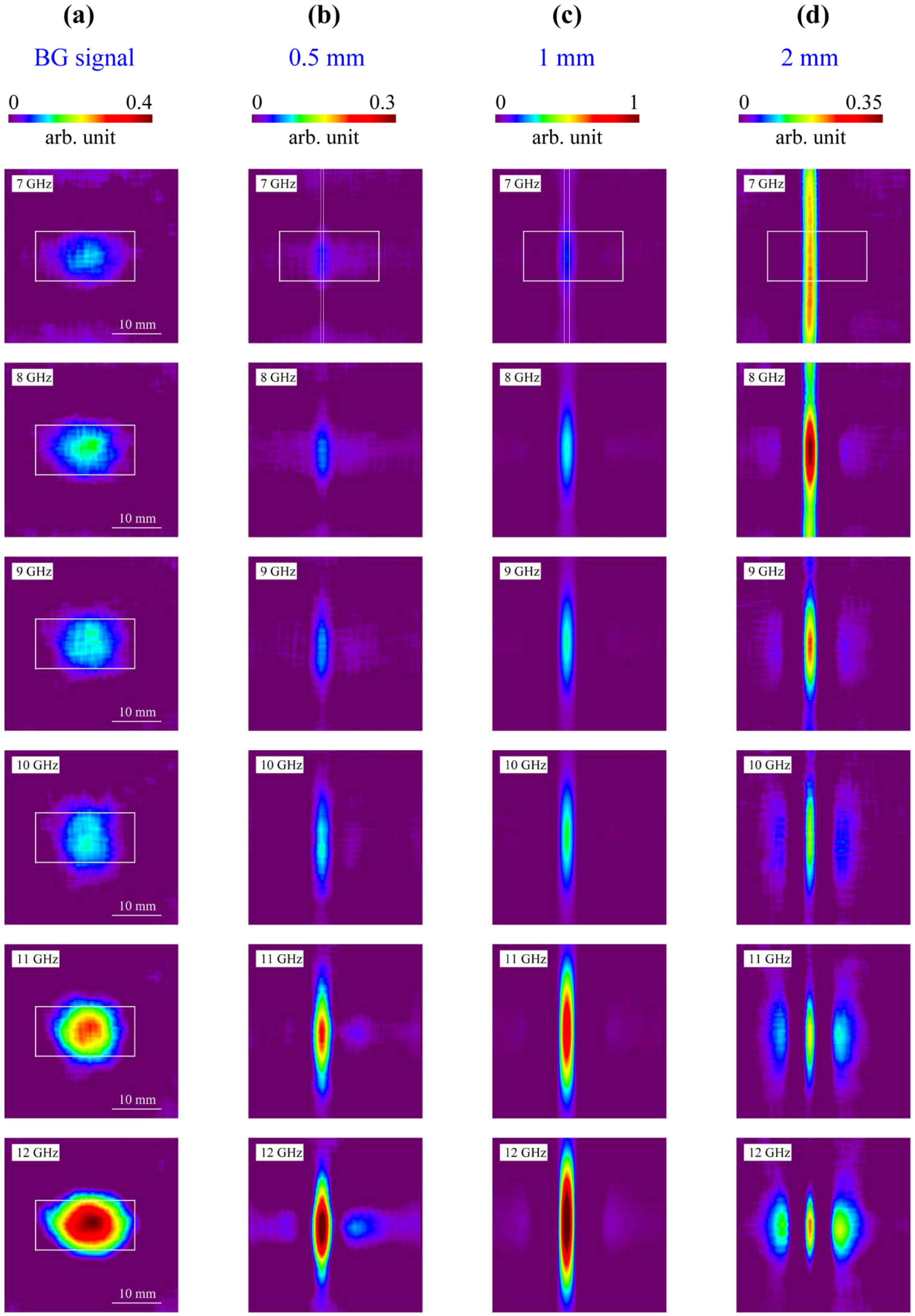
(a)
BG signal
0
0.4
arb. unit
(b)
0.5 mm
0
0.3
arb. unit
(c)
1 mm
0
1
arb. unit
(d)
2 mm
0
0.35
arb. unit
7 GHz
8 GHz
9 GHz
10 GHz
11 GHz
12 GHz
10 mm

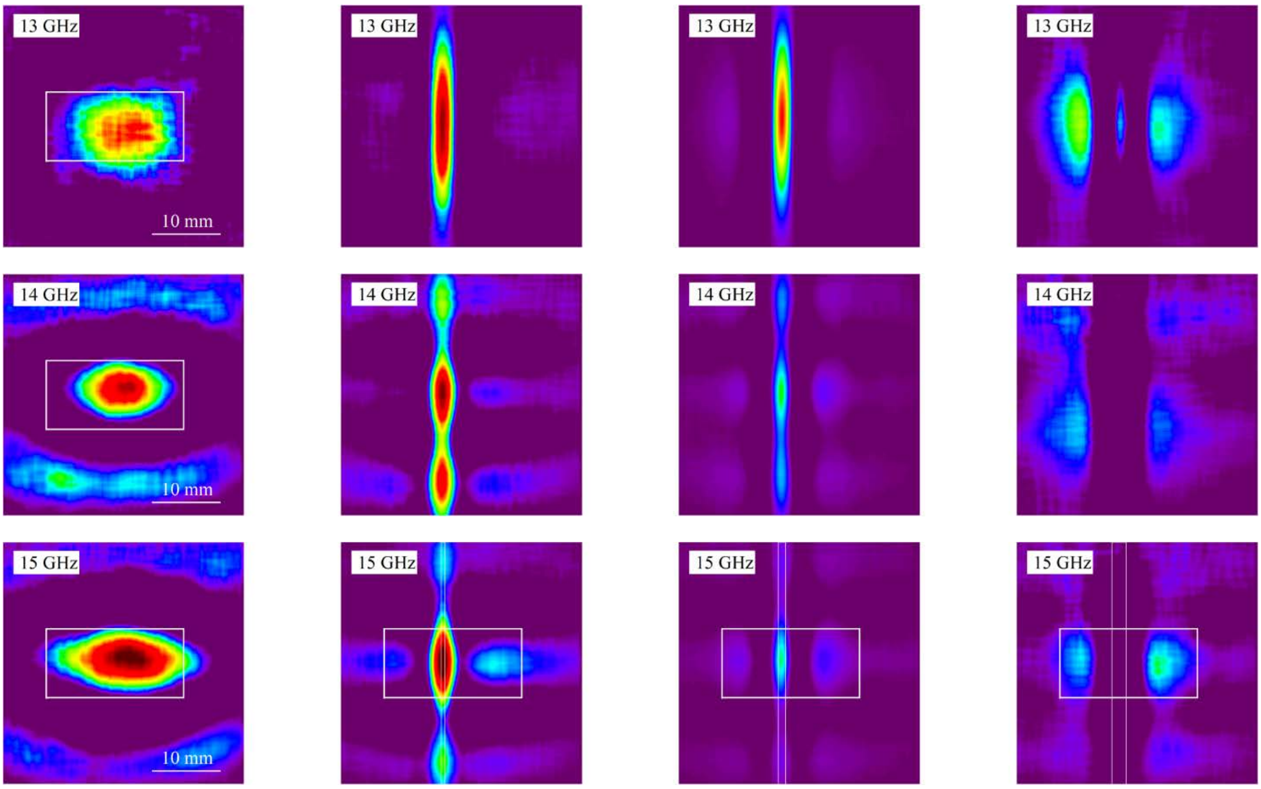

Supplementary figure S6. Visualized H-MWNF distributions at different microwave exposure frequencies for (a) waveguide patterns without sample, and with various tube inner diameters (b) 0.5 mm, (c) 1 mm, and (d) 2 mm filled with DI water. The images for 7 GHz and 15 GHz show the orientation and location of rectangular waveguide and tube. Size of each image is 35 mm × 35 mm.

Figure S7 shows the changes of the average intensity values of the H-MWNF distribution dependence on the microwave exposure frequency.

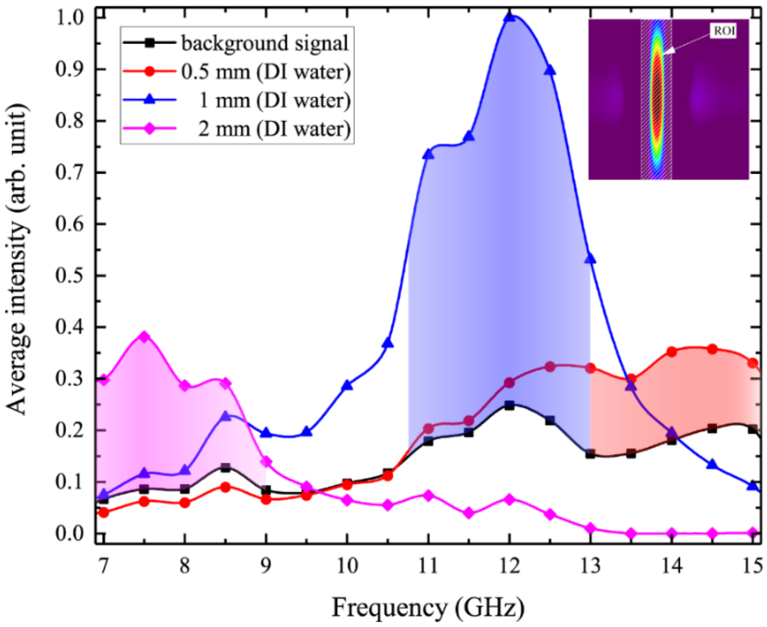

Supplementary figure S7. The changes in the average intensity of the H-MWNF distribution of BG signal (black) and DI water when tube diameter was 0.5 mm (red), 1 mm (blue), and 2 mm (pink) depends on the frequency. The averaged value was calculated by choosing the region of interest (with the same surface area) only around the tube shown in the inset figure. The highlighted regions show the signal difference in the frequency range where the response is stronger for each tube diameter.

Relevant experiments were performed to characterize and compare DI water, glucose (50 mg/ml), and NaCl (50 mg/ml) aqueous solutions through the H-MWNF intensity changes in the same frequency range. Data in Fig. S8 showed that the intensity peaks for the three were similarly detected at 12 GHz without any significant shifts for the solute compounds.

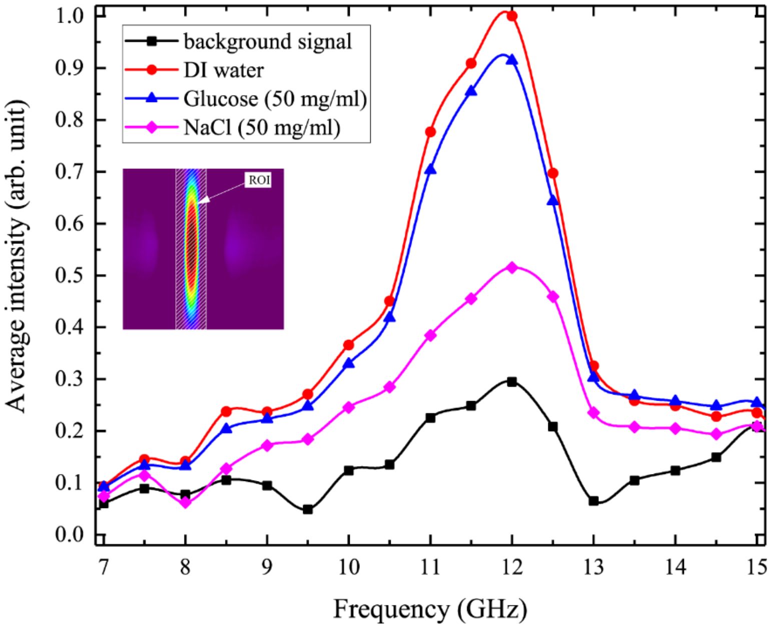

Supplementary figure S8. The changes in the average intensity of the H-MWNF distribution of BG signal (black), DI water (red), glucose (blue), and NaCl (pink) on the frequency. Each solute concentration for glucose and NaCl was 50 mg/ml. The averaged value was calculated by choosing the region of interest (with the same surface area) only around the tube shown in the inset figure.

REFERENCES

1. Babajanyan, A., Kim, J., Kim, S., Lee, K. & Friedman, B. Sodium chloride sensing by using a near-field microwave microprobe. *Appl. Phys. Lett.* **89**, 1–4 (2006).
2. Kim, J., Babajanyan, A., Hovsepyan, A., Lee, K. & Friedman, B. Microwave dielectric resonator biosensor for aqueous glucose solution. *Rev. Sci. Instrum.* **79**, 1–4 (2008).
3. Bababjanyan, A. *et al.* Real-time noninvasive measurement of glucose concentration using a microwave biosensor. *J. Sensors* **2010**, (2010).
4. Odabashyan, L. *et al.* Real-Time Noninvasive Measurement of Glucose Concentration

Using a Modified Hilbert Shaped Microwave Sensor. *Sensors* **19**, 5525 (2019).

5. Liu, C. *et al.* Dielectric properties and microwave heating characteristics of sodium chloride at 2.45 GHz. *High Temp. Mater. Process.* **32**, 587–596 (2013).
6. Gabriel, C., Gabriel, S., Grant, E. H., Halstead, B. S. J. & Michael P Mingos, D. Dielectric parameters relevant to microwave dielectric heating. *Chem. Soc. Rev.* **27**, 213–223 (1998).
7. Liebe, H. J., Hufford, G. A. & Manabe, T. A model for the complex permittivity of water at frequencies below 1 THz. *Int. J. Infrared Millimeter Waves* **12**, 659–675 (1991).
